# A follow-up study of the fate of small asymptomatic deep venous thromboses

**DOI:** 10.1186/1477-9560-8-4

**Published:** 2010-02-12

**Authors:** Stefan Rosfors, Lena M Persson, Gerd Lärfars, Lasse J Lapidus

**Affiliations:** 1 Department of Clinical Science and Education, Karolinska Institutet, Södersjukhuset, SE-118 83 Stockholm, Sweden; 2 Department of Clinical Physiology, Södersjukhuset, SE-118 83 Stockholm, Sweden; 3 Department of Internal Medicine, Södersjukhuset, SE-118 83 Stockholm, Sweden; 4 Department of Orthopedics, Södersjukhuset, SE-118 83 Stockholm, Sweden

## Abstract

**Background:**

Postoperative asymptomatic deep venous thromboses (ADVT) can give rise to posttthrombotic syndrome (PTS), but there are still many unresolved issues in this context. For example, there is a lack of knowledge regarding the fate of small ADVT following minor orthopedic surgery. This follow-up study evaluates postthrombotic changes and clinical manifestations of PTS in a group of patients with asymptomatic calf vein DVT after surgery for Achilles tendon rupture.

**Methods:**

Forty-six consecutive patients with distal ADVT were contacted and enrolled in a follow-up consisting of a single visit at the hospital at a mean time of 5 years postoperatively, including clinical examination and scoring, ultrasonography and venous plethysmography. All patients had participated in DVT-screening with colour duplex ultrasound (CDU) 3 and 6 weeks postoperatively and 80% of them were treated with anticoagulation.

**Results:**

With CDU postthrombotic changes and deep venous reflux were detected at follow-up in more than 50% of the patients, more commonly in somewhat larger calf DVT:s initially affecting more than one vessel. However, only about 10% of the patients had significant venous reflux according to venous plethysmography. No patient had plethysmographic evidence of remaining outflow obstruction, but presence of postthrombotic changes shown with CDU negatively influenced venous outflow capacity measured with plethysmography. A clinical entity of PTS was rarely found and occurred only in two patients (4%) and then classified by Villalta scoring as of mild degree with few clinical signs of disease. Distal ADVT:s detected in the early postoperative period (3 weeks) showed DVT-progression in 75% of the limbs that were still immobilized and without anticoagulation.

**Conclusions:**

Asymptomatic postoperative distal DVT:s following surgery for Achilles tendon rupture have a good prognosis and a favourable clinical outcome. In our material of 46 patients the general appearance of the clinical entity of PTS at 5 years follow-up was low (<5%). Morphological and functional abnormalities were mainly seen in those patients that initially had somewhat larger distal DVT:s involving more than one deep calf vein segment.

## Background

Deep venous thrombosis (DVT) is a common cause of morbidity after orthopedic surgery, both in the shorttime period as pulmonary embolism and for longterm development of postthrombotic syndrome (PTS) [1]. The majority of postoperative DVT:s are small and asymptomatic, nevertheless after major orthopedic surgery asymptomatic DVT (ADVT) can give rise to PTS in around 20% of the patients [2,3]. Therefore, different thromboprophylactic regiments are routinely used in connection with major orthopedic surgery, and during recent years also in increasing frequency with minor orthopedic procedures that involves immobilization of the lower limb. However, the need and the effect of thromboprophylaxis for the latter are not well-established. In general, there is a lack of knowledge regarding the fate of ADVT following minor orthopedic surgery, especially for small ADVT limited to calf veins.

Earlier, contrast phlebography was mandatory to diagnose ADVT and therefore there were limited possibilities for follow-up studies. Technical improvement of the ultrasonographic equipments has changed this so that colour duplex ultrasound (CDU) now has the ability to diagnose ADVT, even those limited to calf veins [4]. This also gives opportunity to study the fate of small ADVT and the development of postthrombotic changes by repeated noninvasive examinations.

The present study reports ultrasonographic findings of ADVT in a group of patients operated for Achilles tendon rupture and a follow-up of these patients after approximately 5 years. The study focuses on detection of postthrombotic changes following calf vein DVT:s and the hemodynamic consequences of these measured with venous plethysmography, and evaluation of the clinical manifestations of PTS in this group of patients.

## Methods

### Study population and design

One-hundred-five consecutive patients were enrolled in a prospective study of postoperative DVT after surgical treatment for Achilles tendon rupture between November 2001 and May 2004. Patients were randomized to double-blinded treatment with either placebo or dalteparin 5.000 U Fragmin (Pfizer) once daily for 6 weeks. The initial study protocol involved a carefully performed colour duplex scanning by highly experienced personal for DVT-screening three and six weeks after surgery. The procedure included evaluation of deep veins from the groin to the ankle, including muscle veins. In general, a phlebography was performed or attempted if the duplex scan was abnormal. Based on these diagnostic tests the physician in charge decided whether treatment should be initiated and if the patients at the 3-week visit would fulfil the study protocol or not. The procedures and the acute results are described in a previous report [5].

Altogether 48 patients had abnormal duplex results indicating DVT, at the time of diagnosis there were two proximal and 46 distal DVT:s (= below the popliteal vein). Both proximal DVT:s occurred in the placebo group and were detected at 3 weeks. The DVT was verified phlebographically in most patients, although it was not attempted in eight of the cases.

Our material consists of the 46 patients with a distal DVT (Table 1). In those cases that were left untreated and where a progress of DVT extension was detected with CDU at 6 weeks, the largest extension is reported in Table 1 (see also Table 2).

**Table 1:** Postoperative asymptomatic distal DVT at ultrasonography, grouped according to size, and distribution of prophylaxis/placebo within each group

	Patients with DVT (*n *= 46)	Prophylaxis/placebo 21/25
1. Isolated calf muscular vein	5 (11%)	2/3
2. One deep calf vein stem †	24 (52%)	13/11
3. Two or more deep calf veins ‡	17 (37%)	6/11

† In 19 patients in a fibular vein, in 5 patients in a posterior tibial vein.

‡ Includes one patient with a muscular vein DVT that during the immobilisation period progressed into calf veins and popliteal vein (see Table 2 - Patient nr 7)

**Table 2:** Colour duplex ultrasound (CDU) findings in 9 patients with distal DVT at 3 weeks, and also investigated 6 weeks postoperatively

Patient	3-week CDU	6-week CDU	Prophylaxis	Warfarin	Phlebography
Nr 1	DVT in two soleus veins	Partial regress, only one soleus vein	Placebo	Started at 3 weeks	Negative, no DVT

Nr 2	DVT in one fibular vein	Unchanged from 3 weeks	Dalteparin	No secondary prophylaxis	Initially interpreted as negative, positive at secondary evaluation

Nr 3	DVT in one soleus vein	Progress into gastrocn and fibular veins	Dalteparin	Started at 6 weeks	Positive, performed at 6 weeks

Nr 4	DVT in one soleus vein	Unchanged from 3 weeks	Placebo	No secondary prophylaxis	Initially interpreted as negative, positive at secondary evaluation

Nr 5	DVT in one gastrocn vein	Progress into one more gastrocn vein	Placebo	No secondary prophylaxis	Not performed

Nr 6	DVT in soleus vein	Progress into two fibular veins	Placebo	Started at 6 weeks	Positive, performed at 6 weeks

Nr 7	DVT in two gastrocn veins	Progress into popliteal and calf veins	Placebo	Started at 6 weeks	Not performed

Nr 8	DVT in one soleus vein	Progress into two fibular veins	Dalteparin	Started at 6 weeks	Positive, performed at 6 weeks

Nr 9	DVT in one posterior tibial vein	Progress into two fibular veins	Dalteparin	Started at 6 weeks	Negative at 3 weeks, not performed at 6 weeks

Thirty-four of the 46 patients with duplex-diagnosed DVT were treated with warfarin for three months. One patient was treated for six months and two patients received only low-molecular-weight heparin for less than three months. Nine patients received no secondary prophylaxis, either because of soleus vein thrombosis that were considered not to need treatment or in some cases because the phlebography initially was interpreted as negative and a DVT was diagnosed later at a standardized evaluation according to the study protocol.

The follow-up consisted of a single visit at the hospital at a mean time of 5 years postoperatively (range, 43 to 71 months), including clinical examination and scoring, ultrasonography and venous plethysmography. The study was approved by the local ethics committee and conducted according to the declaration of Helsinki. All patients gave their written informed consent prior to their inclusion.

### Clinical and laboratory investigations at 5-years follow-up

Symptoms and clinical findings were rated according to the Villalta score. For clinical symptoms, the patients were asked to rate the subjective severity of each of five symptoms (pain, cramps, heaviness, pruritis and paresthesia), and for clinically objective signs the examinator evaluated the severity of each of six signs (edema, redness, skin induration, hyperpigmentation, venous ectasia and pain on calf compression). Both symptoms and objective signs were rated on a scale from 0 (absent) to 3 (severe) and the points were summarized for a total Villalta score where a score of ≥ 5 is used to indicate presence of PTS. Commonly used subcategories are: 0-4 absent or minimal PTS, 5-9 mild PTS, 10-15 moderate PTS and a score of ≥ 15 severe PTS [6,7]. Clinical classification was accomplished using the C-classes of the CEAP score for clinical appearance of venous disease: C0 = no visible or palpable signs of venous disease, C1 = telangiectases or reticular veins, C2 = varicose veins, C3 = edema, C4 = skin changes, C5 = healed ulceration and C6 = active ulceration [8].

CDU was performed using a colour-flow duplex imager (Siemens Acuson Sequoia™ 512 Ultrasound System, Mountain View, CA, USA). A 6 MHz linear-array imaging transducer was used in conjunction with pulsed and colour-flow Doppler equipment. All examinations included both limbs and were performed by a sonographer without knowledge of the results of the previous duplex analysis. The patients were placed in a supine position for scanning of veins above the knee and in a sitting position for scanning of veins at and below the knee. The following venous segments were evaluated for each patient: common femoral, femoral (mid-thigh), popliteal, posterior tibial, fibular, gastrocnemius, soleus and great saphenous (thigh and calf). Vessel wall abnormality (irregularities, wall thickening, non-compressibility, reduced or occluded lumens) was used to assess postthrombotic changes. Valvular function of all veins was evaluated by distal manual compression. A reflux duration longer than 0.5 s was considered significant [9,10].

Venous function tests were performed using computerized strain-gauge plethysmography. The procedure comprised static measurement of venous outflow capacity and dynamic measurement of volume change after muscular exercise, using a computerized mercury strain-gauge plethysmograph (S.I. Veintest 2, Sels Instruments N.V., Vorselaar, Belgium). Using strain-gauge wires around each calf, venous emptying (VE, ml/100 ml × min) was measured and expressed as the outflow rate during the first second after release of venous occlusion by thigh cuffs inflated to a pressure of 60 mmHg [11]. A low VE (< 50 ml/100 ml × min) indicates a significant venous outflow obstruction [12,13]. For dynamic measurements, strain-gauge wires were applied just proximal to the malleolus and volume changes were measured during and after 15 knee bends. Half-refilling time (T 1/2, sec) was measured from the refilling curve after exercise. A short T 1/2 (< 7 sec) indicates venous reflux [14].

### Data analysis and statistical methods

Data are presented as mean and SD. Differences between means were tested for significance using paired and unpaired two-sided Student's t-tests. Differences between proportions were analyzed using chi-square tests. Correlation analyses were used to characterize relationships between variables. Statistical significance was assumed at *P *< 0.05. The statistical analyses were performed using Statistica 8.0 (StatSoft Inc., Tulsa, OK, USA).

## Results

There were 36 males and 10 females with a mean age of 41 years (range 23-60). Their body mass index was 26 (SD 3) and did not change from time of diagnosis to the follow-up examination. The side of the operation was equally distributed (21 right/25 left). None of them had a history of previous DVT, but five patients had heredity for venous thromboembolism. Two patients experienced a new DVT, in the contralateral limb, during the follow-up period. No patient had pulmonary embolism.

### Thrombus evolution at 3 and 6 weeks

Thirty of the 46 distal DVT:s were diagnosed at 3 weeks and the remaining 16 at the 6-week control. Of the 30 DVT:s diagnosed early, 18 (60%) occurred in patients in the placebo group and 12 (40%) occurred in patients belonging to the group receiving pharmacological prophylaxis (p = 0.9). In other words, of those with DVT in the placebo group there was an overweight of early occurring thrombus (72% at 3 weeks), whereas patients receiving prophylaxis had a more equal distribution between time for thrombus development (57% at 3 weeks and 43% at 6 weeks).

Nine of the patients underwent both 3- and 6-weeks ultrasound examination, despite a positive finding at 3 weeks. Eight of these patients received no secondary prophylaxis from 3 to 6 weeks and in six of them the thrombus progressed into more proximal and larger deep veins (Table 2). As can be seen in Table 2 progression mainly concerned muscle vein DVT, since five of the six muscle vein DVT:s that were left untreated during continued immobilisation showed thrombus progression.

### Colour duplex ultrasound at 5 years follow-up

Postthrombotic changes were seen in 25 of the 46 (54%) operated limbs with postoperative distal DVT, in all cases limited to the distal veins. Ten of these patients had at least one still occluded distal vein segment. Postthrombotic changes were also seen in two contralateral limbs, in one case involving both proximal and distal veins. This patient had had a new symptomatic DVT after a knee operation during the follow-up. The other patient with contralateral postthrombotic changes had undergone an uncomplicated operation of an Achilles tendon rupture also in this leg three years following the initial event.

Deep venous reflux was found in 24 of the 46 (52%) operated limbs and also in four contralateral limbs. Twenty-three of the operated limbs had reflux isolated to distal segments, in six limbs involving both posterior tibial veins and fibular veins and in 17 limbs only either of these. One patient had reflux in the popliteal vein and in posterior tibial and fibular veins. Two operated limbs had deep venous reflux without any postthrombotic changes; one fibular and one posterior tibial vein segment, and three limbs had postthrombotic changes without any deep venous reflux. Deep venous reflux in the contralateral limbs regarded in one case three segments (popliteal, posterior tibial, fibular) in a patient with a recent symptomatic DVT (see above), and in the remaining three cases both posterior and fibular reflux, isolated posterior tibial reflux and isolated fibular vein reflux. Reflux in the great saphenous vein was seen in 22 operated limbs and in 14 contralateral limbs (n.s.).

The relation between initial thrombus segment and localization of postthrombotic changes at follow-up is shown in Table 3. In four patients we found postthrombotic changes in deep veins that initially were free from thrombus, this concerned in three cases fibular DVT with postthrombotic changes at follow-up seen also in posterior tibial veins and vice versa in one case (one of these were in a patient who did not receive secondary prophylaxis). Deep venous reflux at follow-up was more commonly found in initially large DVT:s (Group 3 in Table 1) than in initially small ones limited to only one deep calf vein (Group 2 in Table 1); 13/17 (76%) vs.11/24 (46%) p < 0.05 (see Table 1). The same was found for presence of postthrombotic changes, but this difference did not reach statistical significance; 71% vs. 54%.

**Table 3:** Relation between initial thrombus vessel and presence and location of postthrombotic changes as seen by ultrasonography at 5 years follow-up

	No post-thrombotic changes	Posttrombotic changes		
		
		Fewer segments	Same segments	New segments
**Initial DVT vein segment:**				
Muscular veins (n = 5)	5			
Post tibial veins, PTV (n = 7)	3		3	1
Fibular veins, F (n = 27)	12		12	3
PTV + F (n = 7) †	1	5	1	

† Includes one patient with a muscular vein DVT that during the immobilisation period progressed into calf veins and popliteal vein (see Table 2 - Patient nr 7)

### Venous plethysmography at 5 years follow-up

Plethysmographic results for operated and contralateral limbs are shown in Table 4, there was no significant difference between the limbs. In the operated limbs, there was a significant negative relationship between presence of postthrombotic changes and rate of venous emptying (VE; r= -0.36, p = 0.012). However, there was no significant relationship between T 1/2 and presence of deep and/or superficial venous reflux. Four patients had a T 1/2 < 7 sec indicating significant venous reflux, all these had deep venous reflux demonstrated with CDU.

**Table 4:** Results from venous plethysmography at 5 years follow-up in limbs with postoperative distal DVT and in contralateral limbs.

	VE (ml/100 ml × min)	T 1/2 (sec)
Contralateral limbs (n = 46)	107 (23)	16 (8)
DVT limbs, all (n = 46)	102 (24)	17 (9)
1. Isolated calf muscular vein (n = 5)	102 (12)	15 (6)
2. One deep calf vein stem (n = 24)	103 (27)	19 (10)
3. Two or more deep calf veins (n = 17)	102 (23)	15 (8)

Mean and (SD); VE, venous emptying; T 1/2, half-refilling time

### Clinical examination at 5 years follow-up

The Villalta symptom score ranged from 0 to 7 (median 0) and the score for clinical signs ranged from 0 to 2 (median 0). Twenty-eight patients (61%) scored 0 for symptoms, 32 patients (70%) scored 0 for clinical signs and 22 patients (48%) had a total Villalta score of 0. The total Villalta PTS score ranged from 0 to 8 (median 1) and three patients (7%) had a score ≥ 5 indicating presence of PTS. All three patients were classified as having mild PTS (a score of 5-9). Regarding clinical classification according to CEAP 29 patients were classified as C0, 10 patients as C1, six patients as C2 and one patient as C3 because of slight edema.

Two of the three patients with total Villalta score ≥ 5 had DVT:s affecting more than one vessel and with remaining postthrombotic changes in combination with deep venous reflux. Their CEAP-classes were 2 and 3, respectively. The third patient had no morphological or physiological abnormalities in the venous system at follow-up and the CEAP-class was 1. His Villalta score of 8 consisted mainly of a symptom score of 7 and no real clinical signs of venous disease.

## Discussion

The present study regards a consecutive series of 46 patients with small ADVT initially limited to calf veins diagnosed with CDU following surgery for Achilles tendon rupture, and the ultrasonographic appearance of these thrombi in the acute phase and at 5 years follow-up. Postthrombotic changes and deep venous reflux were detected at follow-up in more than 50% of the patients. In this respect, initial thrombus size was of importance since these abnormalities more commonly occurred in calf DVT:s initially affecting more than one vessel. Moreover, presence of postthrombotic changes negatively influenced venous outflow capacity measured with venous plethysmography. However, the general appearance of the clinical entity of PTS was low (around 5%).

There is still a debate whether the proper treatment of isolated calf DVT is anticoagulation or serial follow-up to identify propagation into proximal veins [15,16]. Regarding asymptomatic postoperative calf DVT:s the situation is even more complex since they will not be detected unless a diagnostic screening procedure is performed. Once detected, either of the two management strategies above is adopted. There are no firm results in the literature regarding the risk of withholding treatment of distal ADVT:s and the present study was not designed for that purpose. There were only nine patients in our series that did not receive anticoagulation, they were left untreated in a nonrandomized fashion. However, something that our study demonstrated was that ADVT that are left untreated and with the limb still immobilized will progress and propagate into larger veins in a substantial number of cases. Thus, in our series propagation was seen in six of eight limbs with DVT at 3 weeks that was left without further treatment but still immobilized (Table 2).

The randomized series from where this cohort of patients is taken could not demonstrate a beneficial effect of pharmacological prophylaxis regarding incidence of postoperative ADVT [5]. What could be seen in this further evaluation of the patients with ADVT diagnosed with CDU was that prophylaxis tended to delay the occurrence of DVT compared with the DVT:s occurring in the group receiving placebo. This was even more evident when also proximal DVT:s are considered, since both these occurred early and in the placebo group. This possible effect of prophylaxis is potentially beneficial since the limbs with DVT occurring later will for a shorter period be immobilized and thus reduces a substantial trigger for thrombus progression resulting in smaller DVT:s, and from a theoretical point less frequency of pulmonary embolism.

Postthrombotic changes and deep venous reflux were more common in patients with large than in small DVT:s. This is theoretically reasonable and the validity of our findings with CDU are supported by the fact that presence of postthrombotic changes negatively influenced venous outflow capacity measured with plethysmography. Similar to other reports concerning symptomatic calf DVT, we found the fibular vein to be the most common DVT site [17,18]. In contrast to what Masuda et al found at follow-up [18], we observed a rather high degree of deep venous reflux and also still-occluded vein segments. Improvements in the ultrasonographic technique during the last decade with increased diagnostic ability might be part of this discrepancy. One further observation was presence of postthrombotic changes and deep venous reflux at follow-up found in veins not primarily affected by DVT, probably representing thrombus progression during anticoagulation or formation of silent re-thrombosis at later occasions. Both thrombus extension and late occurring reflux in veins not involved in DVT are earlier described for symptomatic DVT:s [18-21].

None of the patients had a remaining venous outflow obstruction (= VE < 50 ml/100 ml × min), the lowest values were found in two patients with postthrombotic changes (58 and 59 ml/100 ml × min, respectively). We found no significant relation between plethysmographic measures of reflux (T 1/2) and presence of reflux demonstrated with CDU. Similar observations are earlier described using another type of plethysmographic evaluation [22]. This lack of relationship probably reflects a combination of the fact that CDU also detects "normal reflux" in some individuals that have no relation to earlier DVT, occurrence of postthrombotic reflux without hemodynamic importance, and the rather high variability of these measurements obtained during exercise [23]. A more distinct and accurate interpretation of reflux shown with CDU is to consider deep venous reflux to be of real hemodynamic importance only in cases with a short T 1/2, as we found in only four of our 24 (17%) DVT-limbs with reflux in deep veins.

We used the Villalta score to assess PTS and as suggested by others a total score of ≥ 5 indicates presence of PTS [24,25]. The Villalta score is in our opinion the best validated clinical scale to diagnose PTS, a disadvantage when comparison with other studies is that a number of them does not use validated scales and the definition of PTS varies [26,27]. Nevertheless, it seems from the literature as the incidence of PTS following ADVT might have decreased during the last decades from approximately 20-30% to around 10% [2,3,28,29]. Besides methodological differences an explanation can be better prophylactic regiments and earlier mobilization resulting in smaller DVT:s with better prognosis as suggested by our results. Another factor that might have changed over time is use of compression stockings, where increased use probably reduces the risk of PTS [29-31].

Besides a validated clinical scale, we think it is necessary to use methods that can demonstrate residual morphological and functional abnormalities in this group of patients. In our study three patients (7%) had a Villalta score ≥ 5 indicating presence of PTS. However, in contrast to the other two patients one of these had normal findings with CDU and plethysmography and we suggest that his subjective symptoms are caused by musculoskeletal problems that might be related to surgery and it is probably so that the true incidence of PTS in our material is as low as 4% (2/46).

## Conclusions

Asymptomatic postoperative distal DVT:s have a good prognosis and a favourable clinical outcome. At 5 years follow-up of 46 patients with distal ADVT:s morphological and functional abnormalities were mainly seen in those patients that initially had somewhat larger DVT:s involving more than one deep calf vein. In our material the clinical entity of PTS was rarely found and could be documented only in two patients (4%) and then classified as of mild degree with few clinical signs of disease. Distal ADVT:s detected in the early postoperative period (3 weeks) showed DVT-progression in 75% of the limbs that were still immobilized and without anticoagulation.

## Abbreviations

ADVT: asymptomatic deep venous thrombosis; CDU: colour duplex ultrasound; VE: venous emptying (plethysmographic index of outflow capacity); PTS: postthrombotic syndrome

## Competing interests

The authors declare that they have no competing interests.

## Authors' contributions

LJL included the patients in the initial randomised study. SR, LMP, GL and LJL together designed the follow-up study. SR and LMP retrieved the data and performed the statistical analyses. SR draftet the manuscript, with contributions from LMP. All authors read and approved the final manuscript.
